# Water window ptychographic imaging with characterized coherent X-rays

**DOI:** 10.1107/S1600577515005524

**Published:** 2015-04-23

**Authors:** Max Rose, Petr Skopintsev, Dmitry Dzhigaev, Oleg Gorobtsov, Tobias Senkbeil, Andreas von Gundlach, Thomas Gorniak, Anatoly Shabalin, Jens Viefhaus, Axel Rosenhahn, Ivan Vartanyants

**Affiliations:** a Deutsches Elektronen-Synchrotron DESY, Notkestrasse 85, 22607 Hamburg, Germany; b Moscow Institute of Physics and Technology (State University), Dolgoprudny, Moscow Region 141700, Russia; c National Research Nuclear University ‘MEPhI’ (Moscow Engineering Physics Institute), Kashirskoe shosse 31, 115409 Moscow, Russia; d National Research Center, ‘Kurchatov Institute’, Kurchatov Square 1, 123182 Moscow, Russia; e Analytical Chemistry – Biointerfaces, Ruhr University Bochum, Universitätsstrasse 150, 44780 Bochum, Germany; f Applied Physical Chemistry, University of Heidelberg, Im Neuenheimer Feld 253, 69120 Heidelberg, Germany; g Institute of Functional Interfaces, Karlsruhe Institute of Technology, Eggenstein-Leopoldshafen, Germany

**Keywords:** ptychography, non-redundant array, water window imaging, diatom

## Abstract

A ptychographical coherent diffractive imaging experiment in the water window with focused soft X-rays at 500 eV is reported. An X-ray beam with high degree of coherence was selected for ptychography at the P04 beamline of PETRA III synchrotron radiation source. The beam coherence was measured with the newly developed non-redundant array method, and a coherence length of 4.1 µm and global degree of coherence of 35% at 100 µm exit slit opening in the vertical direction were determined. A pinhole, 2.6 µm in size, selected the coherent part of the beam that was used to obtain ptychographic reconstruction results of a lithographically manufactured test sample and a fossil diatom. The achieved resolution was 53 nm for the test sample and was only limited by the size of the detector. The diatom was imaged at a resolution better than 90 nm.

## Abstract 2

Water window ptychographic coherent diffractive imaging was demonstrated at the P04 beamline of PETRA III synchrotron radiation source. The beam coherence was characterized with the non-redundant array method.

## 1. Introduction   

Imaging in the water window energy range between the absorption edges of carbon and oxygen at 284 eV and 532 eV yields a high chemical contrast in biological material with its aqueous components (Larabell & Nugent, 2010 ▶). The coherent X-ray diffractive imaging (CXDI) method (Miao *et al.*, 1999 ▶) applied with third-generation synchrotron sources has proven to be a useful tool in structural analysis on the nanoscale (Chapman & Nugent, 2010 ▶; Mancuso *et al.*, 2010*a* ▶; Thibault & Elser, 2010 ▶; Abbey, 2013 ▶; Vartanyants & Yefanov, 2015 ▶). In CXDI no lenses are used and this, in principle, allows the resolution limitations of conventional lens microscopes to be overcome. Reconstruction of an object from measured diffraction patterns requires solving the well known phase problem. Iterative phase retrieval techniques have been successfully employed to solve the phase problem (Fienup, 1982 ▶; Marchesini, 2007 ▶). As a limitation, the CXDI technique requires the sample to be isolated and fully illuminated by the coherent X-ray beam. In order to study extended objects with X-rays and to improve the uniqueness and convergence of the phase retrieval process, ptychographic coherent diffractive imaging (PCDI) was employed (Rodenburg *et al.*, 2007 ▶). PCDI involves scanning of the X-ray beam along the object up to a desired field of view. A certain overlap of the illuminated areas is crucial to succeed with the phase retrieval and the image reconstruction (Bunk *et al.*, 2008 ▶). Conventional CXDI and earlier iterative algorithms for PCDI required precise knowledge of the probe, for example, the X-ray beam intensity profile incident on the object. *A priori* knowledge of the probe in ptychography is no longer necessary with algorithms that retrieve object and probe simultaneously (Thibault *et al.*, 2008 ▶). Moreover, ptychography has become an excellent tool to characterize optical elements such as pinholes (Giewekemeyer *et al.*, 2010*a* ▶), zone plates (Thibault *et al.*, 2008 ▶), focusing mirrors (Kewish *et al.*, 2010 ▶; Wilke *et al.*, 2014 ▶), compound refractive lenses (Schropp *et al.*, 2010 ▶), X-ray waveguides (Giewekemeyer *et al.*, 2010*b* ▶), and effects on the phase of the wavefield in the focus (Dzhigaev *et al.*, 2014 ▶).

Third-generation synchrotron sources provide intense and highly coherent X-rays which are widely used for coherent diffractive imaging on the nanoscale. The degree of coherence may not be constant along the cross section of the X-ray beam (Vartanyants & Robinson, 2003 ▶). We determined the coherence properties of the X-ray beam before performing our PCDI experiment and selected the most coherent part of the beam to avoid degradation of contrast in the diffraction patterns. Partial coherence may cause artifacts in the image reconstructions and limits the spatial resolution (Vartanyants & Robinson, 2001 ▶; Williams *et al.*, 2007 ▶; Whitehead *et al.*, 2009 ▶; Chen *et al.*, 2012 ▶; Thibault & Menzel, 2013 ▶). The most direct strategy to determine the spatial coherence of soft X-rays from synchrotron sources is to perform a set of Young’s double-slit experiments with different separations between the slits (Chang *et al.*, 2000 ▶; Paterson *et al.*, 2001 ▶). At the same time, the approach of coherence characterization by non-redundant arrays (NRAs) recently applied to X-rays (Skopintsev *et al.*, 2014 ▶) offers a fast and reliable method to obtain the spatial coherence properties of synchrotron radiation from a single measurement.

Fossil diatoms are unique monads with a light-weight exoskeleton consisting of silicon dioxide (SiO_2_). They exhibit very fine periodic three-dimensional structures on the nano- and microscale at the same time which can hardly be manufactured by current nano-technology methods. A fossil diatom can thus be used as an X-ray resolution test sample made by nature. Previously, silica shells of fossil diatoms were successfully studied with coherent imaging methods at synchrotrons (Giewekemeyer *et al.*, 2010*a* ▶; Guehrs *et al.*, 2012 ▶) and free-electron laser sources (Mancuso *et al.*, 2010*b* ▶). The diatom investigated in this work belongs to the dominating species of nano-planktonic pennate *Fragilariopsis cylindrus* that is typically found in ice-edge zones in Antarctic waters (Kang & Fryxell, 1992 ▶).

In this paper, we first present the characterization of the spatial coherence of the soft X-ray beamline with an NRA. This is followed by two PCDI measurements in the water window with optimized beam coherence. A lithographically manufactured test pattern of known structure and the fossil diatom are reconstructed as high-resolution amplitude and phase contrast projection images.

## 2. Experiment   

**Figure 1:**
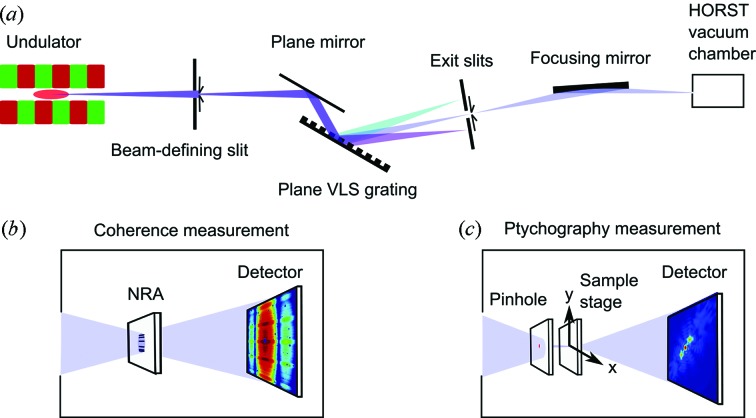
(*a*) Soft X-ray beamline layout. The X-ray scattering vacuum chamber HORST in the coherence measurement setup (*b*) and the ptychography setup (*c*).

The experiment was performed at the soft X-ray beamline P04 (Viefhaus *et al.*, 2013 ▶) at the PETRA III synchrotron radiation facility at DESY in Hamburg. The schematic layout of the beamline is shown in Fig. 1(*a*) ▶. An APPLE-II type helical undulator of 5 m in length with 72 magnetic periods was tuned to deliver photons at an energy of 500 eV which corresponds to a wavelength of λ = 2.5 nm. The beam propagated to the dedicated X-ray vacuum scattering chamber, Holografische Roentgen-Streuapparatur (HORST) (Gorniak & Rosenhahn, 2014 ▶), through several optical elements, including a beam-defining slit (27 m downstream from the undulator), a horizontal plane mirror (35 m) and a monochromator unit consisting of a vertical plane mirror together with a plane varied-line-spacing (VLS) grating (46 m). The VLS grating focused the beam at the exit slit (71 m). A cylindrical mirror (79.1 m) collimated the beam in the horizontal direction [not shown in Fig. 1(*a*) ▶]. An elliptical mirror (78.5 m) focused the beam in the vertical direction to the sample position (81 m). All mirrors were designed to accept a root-mean-square (r.m.s.) beam size of 6σ.

Knowledge of the coherence properties is an important prerequisite for CXDI experiments. For our ptychographic measurements we defined the size of the probe incident on the sample by a pinhole 2.6 µm in diameter. It was etched with a focused ion beam into a 2 µm-thick gold layer supported by a 100 nm thin Si_3_N_4_ membrane.

In both cases of coherence and ptychography measurements the same sample holder of the HORST chamber was used [see Figs. 1(*b*) and 1(*c*) ▶]. It consisted of a closed-loop piezo-electric stage (P-622.1, Physik Instrumente, Germany) to allow horizontal and vertical scanning of the sample relative to the probing X-ray beam with accuracy below 20 nm. The far-field diffraction pattern intensities were measured by a CCD detector (DODX436-BN, Andor Technology Ltd, Belfast, UK). The square detector area of 27.6 mm × 27.6 mm consisted of 2048 × 2048 pixels with a pixel size of 13.5 µm × 13.5 µm.

## 3. Coherence characterization   

### 3.1. Theory   

A brief description of coherence theory is given in the following section to explain how the coherence properties of the beam can be retrieved from a single NRA diffraction pattern (Skopintsev *et al.*, 2014 ▶). In the theory of optical coherence, the statistical properties of the radiation are described by the mutual coherence function (MCF) 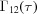 (Mandel & Wolf, 1995 ▶; Goodman, 2000 ▶),
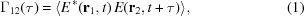
where 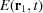 and 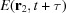 are the field values at positions and times 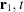 and 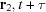, and the angular brackets 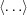 indicate the average over time. The intensity 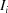 at position 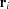 is given by 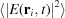. The complex degree of coherence 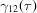 is defined as the normalized MCF,
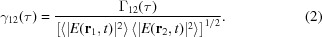
When the time delay τ is much shorter than the coherence time 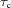, the complex degree of coherence can be approximated by the complex coherence factor (CCF) 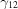 = 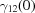 (Goodman, 2000 ▶). To characterize coherence by a single quantity the global degree of coherence ζ is often introduced as (Vartanyants & Singer, 2010 ▶)
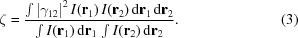
In the frame of the Gaussian Schell model (GSM), which in most cases provides sufficient physical description of the synchrotron radiation, the intensity profile and the CCF are both considered to be Gaussian functions (Mandel & Wolf, 1995 ▶). In this model the partially coherent beam is characterized by the standard deviation σ of the beam size and its transverse coherence length 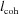. The coherence length is defined as the standard deviation of the modulus of the CCF 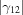. In the frame of GSM, the global degree of coherence ζ from equation (3) can be expressed as (Vartanyants & Singer, 2010 ▶)
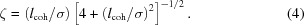
A non-redundant array of apertures can be used to measure the CCF. It was shown (Mejía & González, 2007 ▶; Skopintsev *et al.*, 2014 ▶) that for narrow-bandwidth radiation the intensity 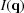 of the far-field interference pattern as a function of the momentum transfer vector 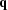 observed in a diffraction experiment with *N* apertures is
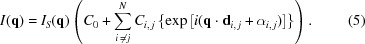
Here 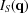 is the diffraction pattern of a single aperture. Individual aperture separations are denoted by 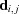 = 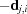 and the relative phases are 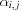 = 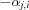. For the analysis of the diffraction pattern from equation (5) its Fourier transform is used,
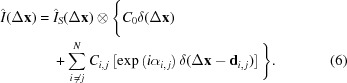
Here 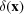 is the Dirac delta function, 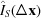 is the Fourier transform of a single aperture diffraction intensity 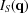 and the symbol 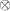 denotes the convolution. The coefficient 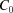 is defined as 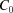 = 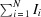, where 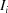 is the intensity incident on the *i*th aperture. The coefficients 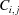 are equal to the MCF values 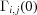 at 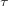 = 0,
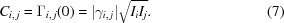
A single peak in equation (6) with its height being equal to 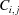 corresponds to each individual aperture separation 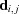. The intensities 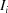, 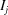 together with peak heights 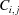 are used to obtain the CCF values from equation (7),
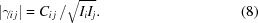



### 3.2. Results of the NRA coherence measurement   

**Figure 2:**
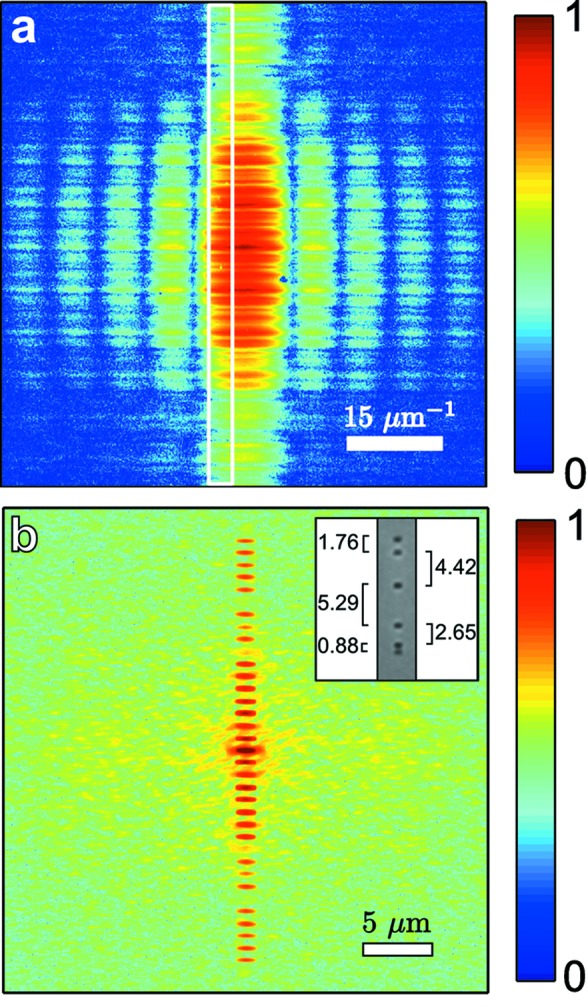
(*a*) Diffraction pattern from a vertically oriented NRA measured at 500 eV and 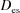 = 100 µm. The white rectangle indicates the area used for the analysis. (*b*) The Fourier transform of the NRA diffraction pattern. Both images are displayed on a logarithmic scale. (Inset) Optical microscope image of the NRA and its aperture separations shown in micrometers.

The coherence properties of the P04 beamline were measured in the focal plane with a single NRA diffraction pattern for each set of beamline parameters. The NRA to detector distance was 50 cm. The analysis was performed using the approach described in the previous section. The coherence values in the vertical direction were determined for 50 µm, 100 µm and 200 µm monochromator exit slit opening 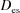. From the monochromator resolving power of 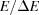 = 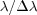 = 6 × 10^3^ the temporal coherence length 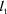 = 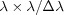 was estimated to be 15 µm. Geometric considerations showed that the maximum optical path length difference in our experiment was 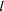 = 0.2 µm in the region used for the coherence analysis. This was much smaller than the temporal coherence length and confirmed our approximation of the complex degree of coherence by the CCF in the previous section. The spatial coherence of the X-ray beam was obtained by measuring the diffraction pattern produced by the NRA at the detector positioned 1 m downstream (see Fig. 1*b* ▶). The NRA consisted of *N* = 6 identical rectangular apertures and was manufactured according to a Golomb ruler (Lam & Sarwate, 1988 ▶). Each aperture was 0.8 µm × 0.25 µm in size. Our NRA was a Golomb ruler of order 6 with each separation between two individual apertures being unique [see inset in Fig. 2(*b*) ▶]. A background-corrected diffraction pattern of the NRA is shown in Fig. 2(*a*) ▶. The Fourier transform 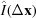 of the measured diffraction pattern from the NRA is presented in Fig. 2(*b*) ▶ and shows 31 well separated peaks.

To determine the CCF we analyzed the area (51 × 2001 pixels) shown as the white rectangle in Fig. 2(*a*) ▶. In this region 51 line scans were used to determine the peak heights 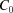 and 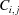. This area corresponds to the part of reciprocal space where the contribution of the high harmonics of X-ray radiation from the undulator is minimal (Skopintsev *et al.*, 2014 ▶).

**Figure 3:**
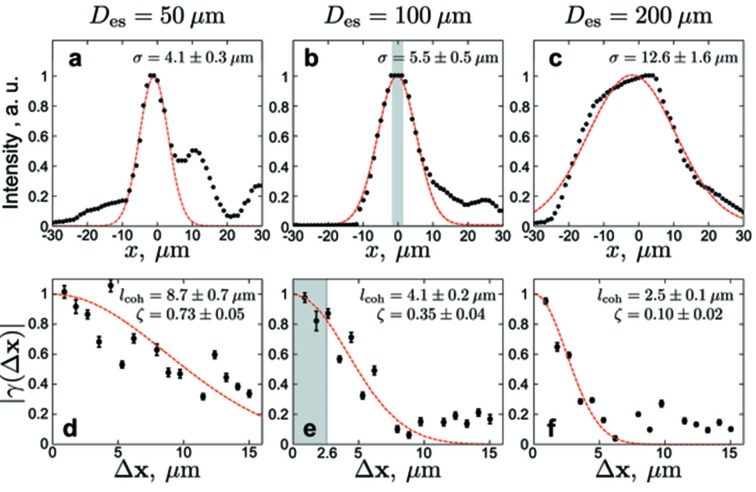
Results of coherence measurement in the vertical direction for three exit slit openings 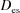 at 500 eV. Black dots indicate measured data and dashed lines represent Gaussian fits. (*a*)–(*c*) Intensity profiles measured with scans of a 1.5 µm pinhole. (*d*)–(*f*) Modulus of the CCF 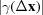. The gray shaded areas in (*b*) and (*e*) indicate the coherent part of the beam selected by the beam-defining 2.6 µm pinhole. The r.m.s. values σ of the beam size obtained from Gaussian fits, the coherence length 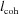, as well as the values of the global degree of coherence ζ determined from equation (4) are also shown. For the Gaussian fits we used the data points up to 9 µm in (*e*) and up to 7 µm in (*f*).

The intensities 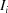 incident on the NRA were determined by a beam profile scan with a pinhole of 1.5 µm diameter and are shown in Figs. 3(*a*)–3(*c*) ▶. For the 50 µm exit slit opening the intensity profile has a narrow peak. The side lobe at the right-hand side of the profile was the result of diffraction from imperfections of the exit slit edges. At the 100 µm exit slit opening the side lobe has almost disappeared because a different section of the exit slit edge was illuminated. At the 200 µm exit slit opening a broad profile is observed without effects from the slit imperfections. The relative difference of the photon flux for each exit slit opening was measured by a photodiode. For the 200 µm slit opening the highest flux was observed. In the case of 100 µm and 50 µm the flux was reduced by a factor of 2.5 and 8, respectively. The flux was expected to be linearly dependent on the exit slit opening. We attributed the deviation to the imperfections and uncertainty of the exit slit positioning system at exit slit openings smaller than 100 µm.

The CCF was obtained from intensities 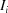 and 51 sets of 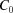, 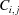. For each set of parameters 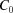, 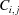 the modulus of the CCF as a function of the NRA aperture separation 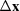 was retrieved using equation (8). In Figs. 3(*d*)–3(*f*) ▶ the averaged CCF values 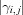 with error bars denoting the standard deviation are presented.

The spatial coherence length 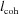 was obtained by the Gaussian approximation 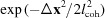 of the averaged CCF 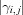. As expected, the coherence length and the global degree of coherence decreased almost linearly with the exit slit opening. The coherence length in the horizontal direction was determined to be 12 µm for all exit slit openings (not shown here).

The characterization of the coherence properties of the X-rays was performed in order to use an optimal exit slit opening for ptychography. In Table 1 ▶ we summarize the photon flux estimated as integrated photon counts at the detector, coherence length and CCF values between two edges of the 2.6 µm pinhole for the three exit slit openings. Although a 50 µm exit slit opening provided the largest coherent fraction of the beam, it also attenuated the beam strongly. For 200 µm opening the CCF reached 0.57 between the edges of the pinhole and thus low contrast in ptychographic data was expected. Finally, for ptychography measurements, the most coherent part of the beam provided by the 100 µm exit slit with the modulus of the CCF 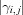 > 0.8 was selected by the 2.6 µm-diameter pinhole [see shaded area in Figs. 3(*b*) and 3(*e*) ▶].

## 4. Ptychography   

In far-field ptychography the diffraction pattern amplitude 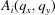 in the detector plane is defined as the Fourier transform of the product of the probe 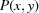 and the projected object function 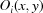,
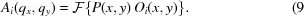
Here *x* and *y* are the coordinates in the sample plane, 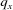 and 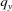 being momentum transfer values and 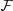 denoting the Fourier transform operator. The index *i* spans along the individual scan position of the probe in the object plane. From the ptychographical reconstruction in transmission geometry (see Fig. 1*c* ▶) one retrieves the complex object function 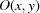 = 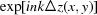 which depends on the wavenumber *k* = 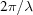, the object thickness 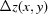 and the complex refractive index *n* = 1 − 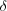 + 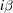. Using the definition for *n* the object function can also be written as 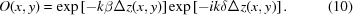
Here the first term with the absorption coefficient β represents the projected amplitude. The exponent of the second term with the refraction coefficient δ is the relative phase shift 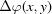 = 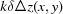. Both terms contain the object specific response to X-ray radiation. First, we examined a strongly scattering tantalum (Ta) test object in the form of a Siemens star (ATN/XRESO-50HC, NTT-AT, Japan). The Siemens star was a lithographically manufactured sample with a smallest feature size of 50 nm. Second, we measured a fossil diatom skeleton dispersed on a silicon nitride (Si_3_N_4_) membrane. With the 2.6 µm pinhole the incident beam was shaped to a highly coherent and circular probe. Both samples were scanned on a rectangular scanning grid with a step size of 800 nm in both *x* and *y* directions perpendicular to the beam propagation axis. The detector was positioned in the far field at a distance of 58 cm downstream from the sample. We applied a dose of approximately 3 × 10^4^ Gy per scan point.

**Figure 4:**
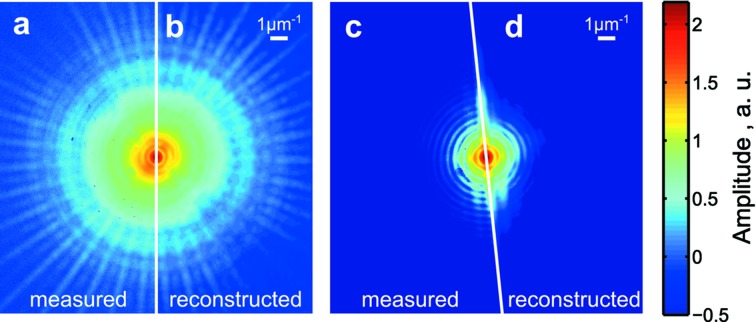
Diffraction images averaged over all positions *i* for the Siemens star (*a*, *b*) and for the diatom (*c*, *d*). Measured (*a*, *c*) and reconstructed diffraction patterns (*b*, *d*) are separated by the white line. The diffraction patterns are displayed on a logarithmic scale.

We implemented the ePIE algorithm (Maiden & Rodenburg, 2009 ▶) to reconstruct both objects.^1^ Initially, the object function was set to zero for all positions *i*. We found this initialization useful because the reconstruction converged faster to a solution than with a randomized initial object function. The probe started with random values in amplitude and phase. The diffracted signal from the Siemens star was visible up to the edges of the detector. The reconstruction succeeded well over the whole diffraction plane [see Figs. 4(*a*) and 4(*b*) ▶]. The diffraction signal from the diatom was comparably weak and reached the noise level already below the edges of the detector [see Figs. 4(*c*) and 4(*d*) ▶]. As a consequence of the limited detector dynamic range, the signal above 7 µm^−1^ was dominated by noise. To reduce the noise contribution in the diatom diffraction patterns we subtracted the mean noise level of each pixel plus four times the standard deviation from this mean that was determined from ten dark images. With this noise removal strategy the noise-related artifacts vanished in the final reconstruction of the diatom.

### 4.1. Reconstruction of the probe function   

**Figure 5:**
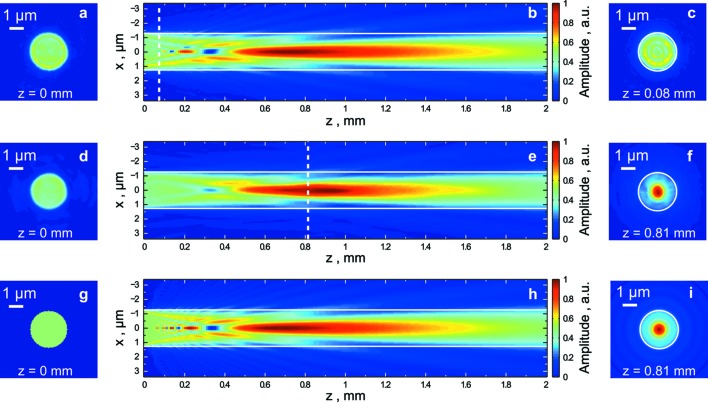
Ptychographic probe function reconstruction from the Siemens star (*a*–*c*) and the diatom (*d*–*f*). A plane wave simulation for propagation of the wavefield from a 2.6 µm pinhole aperture is shown in (*g*, *h*, *i*). Amplitude distribution at the position of the pinhole (*a*, *d*, *g*); distribution of the wavefield amplitude downstream of the pinhole (*b*, *e*, *h*); amplitude distribution of the probe amplitude at the position of the sample (*c*, *f*, *i*) [Siemens star (*c*), and diatom (*f*)]. The simulated amplitude distribution (*i*) is shown at the same *z* position as for (*f*). Dashed white lines in (*b*, *e*) indicate the position of the sample relative to the pinhole at *z* = 0. White circles in (*c*, *f*, *i*) indicate the pinhole size.

We exploited the strength of the ePIE algorithm and reconstructed the complex valued probe along with the object simultaneously. For both samples the complex probe functions were reconstructed and numerically propagated forward and backward and compared with a simulation. This was done to quantify the relative distances between the pinhole and the sample inside the HORST chamber. In Figs. 5(*c*), 5(*f*) and 5(*i*) ▶ we show the reconstructed probes from the experiment together with a calculated ideal probe. The white circles indicate the pinhole size. In Figs. 5(*b*), 5(*e*) and 5(*h*) ▶ we show the amplitudes retrieved by forward and backward Fresnel propagation of the complex valued wavefields. White lines correspond to the pinhole diameter. In Figs. 5(*a*), 5(*d*) and 5(*g*) ▶ we show the beam cross sections in the pinhole plane that resulted from the propagations.

In the simulation shown in Figs. 5(*g*)–5(*i*) ▶ a plane wave was incident on a circular aperture with a diameter of 2.6 µm. The result of the simulation showed excellent agreement with the amplitude of the wavefield distribution obtained from the Siemens star probe especially at short distances behind the pinhole. The only difference was a slightly better contrast for the simulated propagation. A comparison with the wavefield obtained from the diatom showed the same general behavior. However, it was lacking sharp features and high contrast which were both present in the Siemens star and simulated propagation. We attribute this effect to a lower signal produced by the diatom sample.

The pinhole position was determined by calculating the characteristic distance from the pinhole to the pronounced Fresnel diffraction minimum on the optical axis at *z* = 0.34 mm. The probe incident on the Siemens star was determined to be behind the pinhole at a distance of 0.08 mm. It contained a fine structure visible as concentric fringes that is typical for near-field diffraction from a pinhole with a Fresnel number 8.5 [see Fig. 5(*c*) ▶]. We repeated the same procedure and found the diatom sample position at a distance of 0.81 mm behind the pinhole [see Fig. 5(*f*) ▶]. The propagation of the diatom probe wavefield to the position at *z* = 0 produced a well shaped pinhole of 2.6 µm in diameter and practically constant amplitude across the pinhole.

The comparison of the wavefields at the position of the diatom (*z* = 0.81 mm) showed similar amplitude distribution with a beam size of about 1 µm full width at half-maximum (FWHM). Although this was considerably smaller than the pinhole size the probe was still large enough to provide a sufficient overlap between adjacent illumination positions for ptychography.

One interesting result of this investigation is that one has to be careful if a pinhole is used in a ptychography experiment. The beam profile can be significantly different depending on the position behind that pinhole. In our experiment, for example, at *z* = 0.34 mm the beam amplitude was practically equal to zero in the center of the beam; at the same time at the position 0.21 mm behind the pinhole the beam had a sharp and narrow maximum of size 0.2 µm (FWHM) as well as strong shoulders on both sides [see Figs. 5(*b*) and 5(*h*) ▶]. Good conditions for our experiment were in principle at the distances from 0.6 mm to 1.6 mm where the beam had a single pronounced peak and did not contain much amplitude in the side lobes outside of the geometrical pinhole region. A sample position (smaller than 0.1 mm) very close to the pinhole may also be beneficial because phase oscillations result in a structured probe that can improve the ptychographic reconstruction process in some cases (Quiney *et al.*, 2005 ▶; Guizar-Sicairos *et al.*, 2012 ▶; Maiden *et al.*, 2013 ▶).

### 4.2. Test pattern reconstruction   

**Figure 6:**
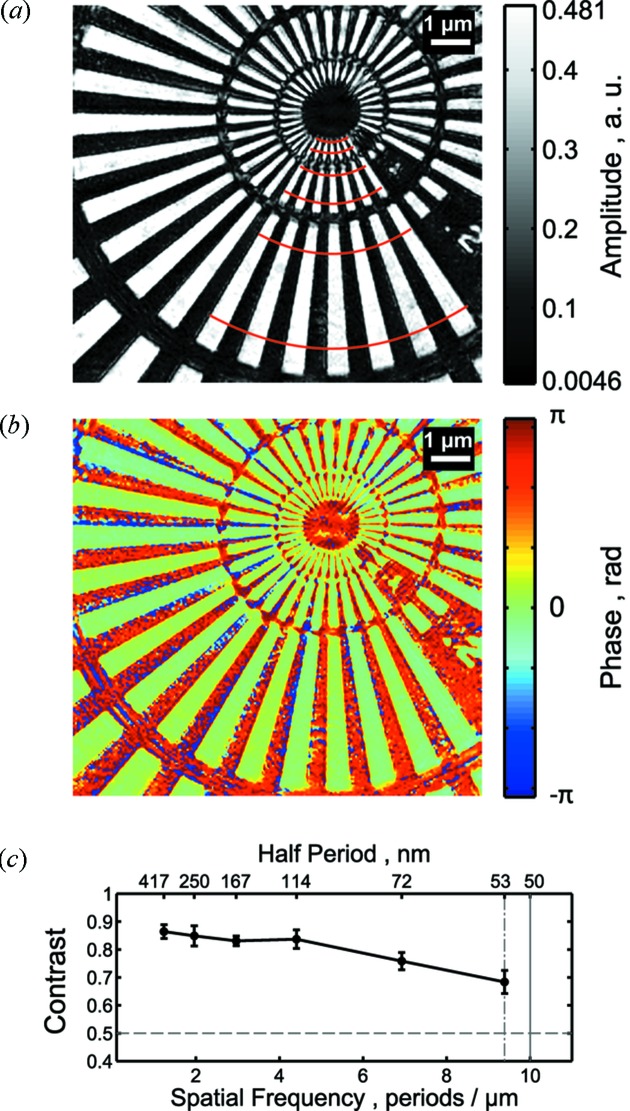
Ptychographic reconstruction of the Siemens star test pattern. (*a*) Amplitude and (*b*) phase image. (*c*) Contrast *C* between high and low transmission as a function of spatial frequency from the angular scans denoted by red lines in the amplitude image.

The dataset from the Siemens star consisted of 132 diffraction patterns (12 horizontal × 11 vertical) that covered an area of 10.4 µm × 9.6 µm around the center of the test pattern. The reconstructed Siemens star amplitude is shown in Fig. 6(*a*) ▶. Black color denotes the strongly absorbing parts made of 270 nm-thick substrate layers from SiC, Si_3_N_4_ and Ru including the test pattern with 500 nm-thick Ta with an expected transmission of 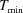 = 4.6 × 10^−3^. In the white areas we expected a high transmission of 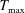 = 0.48 that was defined by the substrate only. These areas had zero phase variation, as can be seen in Fig. 6(*b*) ▶. In the low transmission parts a phase of 6.39 rad was expected; however, undefined phases were observed. That could be explained by the phase value being close to 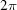 and small fluctuations causing complicated phase wrapping, both of which prevent a reliable and quantitative analysis (Giewekemeyer *et al.*, 2011 ▶).

By performing a series of reconstructions we observed in some cases an incorrect number of lines (other than 36) in the angular direction while the radius of the concentric rings was correct. This effect was also observed previously (Burdet *et al.*, 2014 ▶) and appears in the case of a not accurately known sample-to-detector distance. With our detailed probe function analysis we finally determined an accurate sample-to-detector distance of 578.50 ± 0.01 mm and obtained the excellent reconstruction shown in Fig. 6 ▶.

We used multiple angular line scans to plot the reconstructed amplitude contrast *C* = 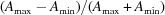 as a function of spatial frequency (see Fig. 6*c* ▶), where 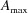 and 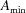 are the averaged maximum and minimum amplitudes of each line-scan, respectively. The error bars represent the standard deviation from the averaged contrast. The contrast decayed slowly from 0.87 at 1 period µm^−1^ to 0.7 at the maximum spatial frequency of 9.4 period µm^−1^, indicating a very good visibility for all spatial frequencies present in the reconstructed object. The maximum spatial frequency corresponds to a half-period length of 53 nm and was equal to the pixel size of the reconstruction. Since the diffracted signal was cut at the detector edges in reciprocal space [see Figs. 4(*a*) and 4(*b*) ▶] the obtained resolution was limited only by the detector size.

### 4.3. Fossil diatom reconstruction   

**Figure 7:**
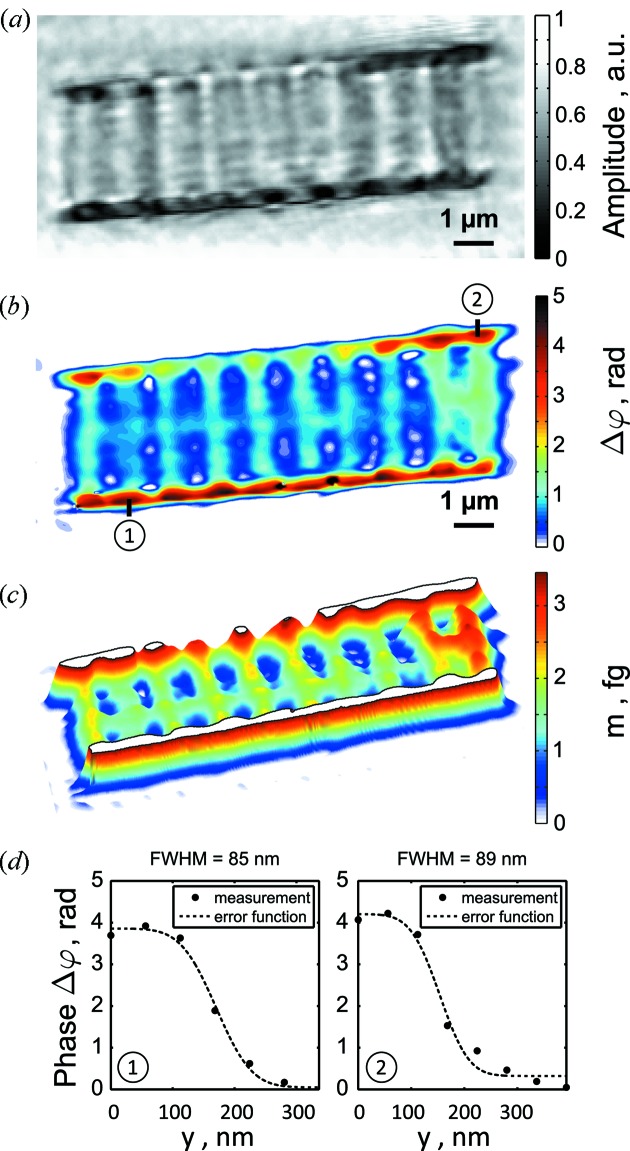
Ptychographic reconstruction of the amplitude (*a*) and the phase (*b*) of the fossil diatom. (*c*) Integrated SiO_2_ mass along the depth of the diatom (see text for further details) and (*d*) FWHM values of two error function fits along the black lines indicated in the phase reconstruction in (*b*).

In the case of the fossil diatom the dataset consisted of 119 diffraction patterns (17 horizontal × 7 vertical) covering an area of 14.4 µm × 6.4 µm. As before in the case of the Siemens star reconstruction, we used our detailed probe function analysis to determine the accurate sample-to-detector distance and in this way avoided the problems of reconstruction of periodic structures. The reconstructed amplitude of the fossil diatom is shown in Fig. 7(*a*) ▶. The reconstructed and unwrapped phase of the sample is shown in Fig. 7(*b*) ▶. The color scheme displays the relative phase shift map 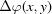 between the substrate (shown in white) and the diatom. Assuming uniform density of SiO_2_ (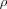 = 2.2 g cm^−3^) we determined the refraction coefficient δ at 500 eV photon energy to be 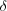 = 1.39 × 10^−3^ (Henke *et al.*, 1993 ▶). This allowed us to convert the relative phase shift 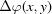 between the substrate and the diatom to the projected material thickness by applying the relation 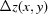 = 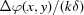. Diatoms of the *Fragilariopsis cylindrus* species have a cylindrically curved shape (see Kang & Fryxell, 1992 ▶). From the two-dimensional projection image we can thus deduce the integrated SiO_2_ mass along the depth of the diatom as 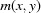 = 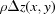. The 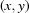 coordinates denote the discrete pixels of the reconstruction with a pixel size of 53 nm × 53 nm. In Fig. 7 ▶ we show the surface plot of the integrated SiO_2_ mass up to a threshold of 3.5 fg (femtogram) to preserve the visibility of the fine structure.

Ten equidistantly spaced ribs are visible with a period of 1 µm. The length and width of each rib was estimated as 3 µm and 250 nm, respectively. The fine structure that appeared in the form of a perforation and which is well pronounced in the amplitude image in Fig. 7(*a*) ▶ had a period of 200 nm in the vertical direction. All the discovered features of the fossil diatom that we investigated here are specific for the species *Fragilariopsis cylindrus* and are comparable with earlier studies (Kang & Fryxell, 1992 ▶; Bertilson *et al*., 2009 ▶).

From line scans, which were extracted from the phase reconstruction, we determined the FWHM values of the fitted error functions [see Fig. 7(*d*) ▶]. Using the FWHM values, the half-period resolution of our ptychographic diatom reconstruction was below 90 nm and, consequently, 30% better than in a previously published paper (Giewekemeyer *et al.*, 2011 ▶).

## 5. Summary   

An experiment for high-resolution ptychographical imaging of extended samples in the water window at 500 eV with highly coherent X-rays from the P04 beamlime at PETRA III has been presented. The spatial coherence was characterized with an efficient NRA method and high coherence of the X-ray beam was demonstrated. The global degree of coherence in the vertical direction varied from 10% to 73% depending on the setting of the exit slit of the monochromator. For ptychographic measurements an optimal exit slit size of 100 µm was used to provide a coherence length in the vertical direction of the focused beam of 4.1 µm and a global degree of coherence of 35% at the sample position. With these settings the most coherent part of the beam was selected by the 2.6 µm pinhole that produced high-contrast diffraction patterns in our ptychographic experiment.

We obtained important knowledge about the fine features of the probe function by the propagation analysis that allowed us to determine the sample position inside the HORST vacuum chamber with high precision. This turned out to be important in imaging periodic samples to avoid the artifacts caused by an inaccurately known sample-to-detector distance. The ptychographic reconstruction of the Siemens star test pattern was obtained up to 53 nm resolution and was limited only by the detector size. In the case of the weakly scattering fossil diatom we obtained the amplitude and phase of the transmission function quantitatively with a resolution better than 90 nm. Finally, we exploited the phase reconstruction and obtained a quantitative integrated material map with details that allowed a comparison with the diatom species reported in the literature.

Ptychography in the water window with its sensitivity for oxygen and carbon contrast is especially promising to image hydrated biological cells (Gorniak & Rosenhahn, 2014 ▶). With the cryo-extension of the HORST vacuum chamber its capability to image extended biological samples on the nanoscale will become feasible. We are also planning to extend our research to three-dimensional ptychography with high spatial resolution (Dierolf *et al.*, 2010 ▶). This will allow us to determine the fine structure of diatoms on the nanoscale in 3D.

## Figures and Tables

**Table 1:** Photon flux , vertical coherence length 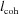 and CCF values for different exit slit openings *D*_es_ of the monochromator

*D*_es_ (m)	(10^6^ photons s^1^)	*l*_coh_ (m)	| 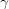 ( 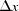 = 2.6m)|
50	1.1	8.7 0.7	0.95 0.01
100	3.5	4.1 0.2	0.82 0.02
200	8.7	2.5 0.1	0.57 0.03

## Footnotes

1 Matlab (version 2013b) code was used for the entire analysis of the ptychography data. All calculations were executed on a graphics processing unit (GPU).

## References

[bb1] Abbey, B. (2013). *JOM*, **65**, 1183–1201.

[bb2] Bertilson, M., von Hofsten, O., Vogt, U., Holmberg, A. & Hertz, H. M. (2009). *Opt. Express*, **17**, 11057–11065. 10.1364/oe.17.011057 19550505

[bb3] Bunk, O., Dierolf, M., Kynde, S., Johnson, I., Marti, O. & Pfeiffer, F. (2008). *Ultramicroscopy*, **108**, 481–487. 10.1016/j.ultramic.2007.08.003 17764845

[bb4] Burdet, N., Morrison, G. R., Huang, X., Shi, X., Clark, J. N., Zhang, F., Civita, M., Harder, R. & Robinson, I. K. (2014). *Opt. Express*, **22**, 10294–10303. 10.1364/OE.22.010294 24921732

[bb5] Chang, C., Naulleau, P., Anderson, E. & Attwood, D. (2000). *Opt. Commun.* **182**, 25–34.

[bb6] Chapman, H. N. & Nugent, K. A. (2010). *Nat. Photon.* **4**, 833–839.

[bb7] Chen, B., Abbey, B., Dilanian, R., Balaur, E., van Riessen, G., Junker, M., Tran, C. Q., Jones, M. W. M., Peele, A. G., McNulty, I., Vine, D. J., Putkunz, C. T., Quiney, H. M. & Nugent, K. A. (2012). *Phys. Rev. B*, **86**, 235401.

[bb8] Dierolf, M., Menzel, A., Thibault, P., Schneider, P., Kewish, C. M., Wepf, R., Bunk, O. & Pfeiffer, F. (2010). *Nature (London)*, **467**, 436–439. 10.1038/nature09419 20864997

[bb9] Dzhigaev, D., Lorenz, U., Kurta, R. P., Seiboth, F., Stankevic, T., Mickevicius, S., Singer, A., Shabalin, A., Yefanov, O. M., Strikhanov, M. N., Falkenberg, G., Schroer, C. G., Feidenhans’l, R. & Vartanyants, I. A. (2014). *J. Phys. Conf. Ser.* **499**, 012020.

[bb10] Fienup, J. R. (1982). *Appl. Opt.* **21**, 2758–2769. 10.1364/AO.21.002758 20396114

[bb11] Giewekemeyer, K., Beckers, M., Gorniak, T., Grunze, M., Salditt, T. & Rosenhahn, A. (2011). *Opt. Express*, **19**, 1037–1050. 10.1364/OE.19.001037 21263642

[bb12] Giewekemeyer, K., Neubauer, H., Kalbfleisch, S., Krüger, S. P. & Salditt, T. (2010*b*). *New J. Phys.* **12**, 035008. 10.1364/OE.18.013492 20588479

[bb13] Giewekemeyer, K., Thibault, P., Kalbfleisch, S., Beerlink, A., Kewish, C. M., Dierolf, M., Pfeiffer, F. & Salditt, T. (2010*a*). *Proc. Natl. Acad. Sci.* **107**, 529–534. 10.1073/pnas.0905846107 PMC2795774 20018650

[bb14] Goodman, J. (2000). *Statistical Optics.* New York: Wiley.

[bb15] Gorniak, T. & Rosenhahn, A. (2014). *Z. Phys. Chem.* **228**, 1089.

[bb16] Guehrs, E., Stadler, A. M., Flewett, S., Frömmel, S., Geilhufe, J., Pfau, B., Rander, T., Schaffert, S., Büldt, G. & Eisebitt, S. (2012). *New J. Phys.* **14**, 013022.

[bb17] Guizar-Sicairos, M., Holler, M., Diaz, A., Vila-Comamala, J., Bunk, O. & Menzel, A. (2012). *Phys. Rev. B*, **86**, 100103.

[bb18] Henke, B. L., Gullikson, E. M. & Davis, J. C. (1993). *At. Data Nucl. Data Tables*, **54**, 181–342.

[bb19] Kang, S.-H. & Fryxell, G. A. (1992). *Polar Biol.* **12**, 609–627.

[bb20] Kewish, C. M., Thibault, P., Dierolf, M., Bunk, O., Menzel, A., Vila-Comamala, J., Jefimovs, K. & Pfeiffer, F. (2010). *Ultramicroscopy*, **110**, 325–329. 10.1016/j.ultramic.2010.01.004 20116927

[bb21] Lam, A. W. & Sarwate, D. V. (1988). *IEEE Trans. Commun.* **36**, 380–382.

[bb22] Larabell, C. A. & Nugent, K. A. (2010). *Curr. Opin. Struct. Biol.* **20**, 623–631. 10.1016/j.sbi.2010.08.008 PMC3268817 20869868

[bb23] Maiden, A. M., Morrison, G. R., Kaulich, B., Gianoncelli, A. & M.; Rodenburg, J. M. (2013). *Nat. Commun.* **4**, 1669. 10.1038/ncomms2640 23575673

[bb24] Maiden, A. M. & Rodenburg, J. M. (2009). *Ultramicroscopy*, **109**, 1256–1262. 10.1016/j.ultramic.2009.05.012 19541420

[bb25] Mancuso, A. P., Gorniak, T., Staier, F., Yefanov, O. M., Barth, R., Christophis, C., Reime, B., Gulden, J., Singer, A., Pettit, M. E., Nisius, T., Wilhein, T., Gutt, C., Grübel, G., Guerassimova, N., Treusch, R., Feldhaus, J., Eisebitt, S., Weckert, E., Grunze, M., Rosenhahn, A. & Vartanyants, I. A. (2010*b*). *New J. Phys.* **12**, 035003.

[bb26] Mancuso, A. P., Yefanov, O. M. & Vartanyants, I. A. (2010*a*). *J. Biotechnol.* **149**, 229–237. 10.1016/j.jbiotec.2010.01.024 20149827

[bb27] Mandel, L. & Wolf, E. (1995). *Optical Coherence and Quantum Optics.* Cambridge University Press.

[bb28] Marchesini, S. (2007). *Rev. Sci. Instrum.* **78**, 011301. 10.1063/1.2403783 17503899

[bb29] Mejía, Y. & González, A. I. (2007). *Opt. Commun.* **273**, 428–434.

[bb30] Miao, J., Charalambous, P., Kirz, J. & Sayre, D. (1999). *Nature (London)*, **400**, 342–344.

[bb31] Paterson, D., Allman, B. E., McMahon, P. J., Lin, J., Moldovan, N., Nugent, K. A., McNulty, I., Chantler, C. T., Retsch, C. C., Irving, T. H. K. & Mancini, D. C. (2001). *Opt. Commun.* **195**, 79–84.

[bb32] Quiney, H. M., Nugent, K. A. & Peele, A. G. (2005). *Opt. Lett.* **30**, 1638–1640. 10.1364/ol.30.001638 16075522

[bb33] Rodenburg, J. M., Hurst, A. C., Cullis, A. G., Dobson, B. R., Pfeiffer, F., Bunk, O., David, C., Jefimovs, K. & Johnson, I. (2007). *Phys. Rev. Lett.* **98**, 034801. 10.1103/PhysRevLett.98.034801 17358687

[bb34] Schropp, A., Boye, P., Feldkamp, J. M., Hoppe, R., Patommel, J., Samberg, D., Stephan, S., Giewekemeyer, K., Wilke, R. N., Salditt, T., Gulden, J., Mancuso, A. P., Vartanyants, I. A., Weckert, E., Schöder, S., Burghammer, M. & Schroer, C. G. (2010). *Appl. Phys. Lett.* **96**, 091102.

[bb35] Skopintsev, P., Singer, A., Bach, J., Müller, L., Beyersdorff, B., Schleitzer, S., Gorobtsov, O., Shabalin, A., Kurta, R. P., Dzhigaev, D., Yefanov, O. M., Glaser, L., Sakdinawat, A., Grübel, G., Frömter, R., Oepen, H. P., Viefhaus, J. & Vartanyants, I. A. (2014). *J. Synchrotron Rad.* **21**, 722–728. 10.1107/S1600577514006857 24971966

[bb36] Thibault, P., Dierolf, M., Menzel, A., Bunk, O., David, C. & Pfeiffer, F. (2008). *Science*, **321**, 379–382. 10.1126/science.1158573 18635796

[bb37] Thibault, P. & Elser, V. (2010). *Annu. Rev. Condens. Matter Phys.* **1**, 237–255.

[bb38] Thibault, P. & Menzel, A. (2013). *Nature (London)*, **494**, 68–71. 10.1038/nature11806 23389541

[bb39] Vartanyants, I. A. & Robinson, I. K. (2001). *J. Phys. Condens. Matter*, **13**, 10593–10611.

[bb40] Vartanyants, I. A. & Robinson, I. K. (2003). *Opt. Commun.* **222**, 29–50.

[bb41] Vartanyants, I. A. & Singer, A. (2010). *New J. Phys.* **12**, 035004.

[bb42] Vartanyants, I. A. & Yefanov, O. M. (2015). *X-ray Diffraction: Modern Experimental Techniques*, pp. 341–384. Pan Stanford Publishing.

[bb43] Viefhaus, J., Scholz, F., Deinert, S., Glaser, L., Ilchen, M., Seltmann, J., Walter, P. & Siewert, F. (2013). *Nucl. Instrum. Methods Phys. Res. A*, **710**, 151–154.

[bb44] Whitehead, L. W., Williams, G. J., Quiney, H. M., Vine, D. J., Dilanian, R. A., Flewett, S., Nugent, K. A., Peele, A. G., Balaur, E. & McNulty, I. (2009). *Phys. Rev. Lett.* **103**, 243902. 10.1103/PhysRevLett.103.243902 20366201

[bb45] Wilke, R. N., Wallentin, J., Osterhoff, M., Pennicard, D., Zozulya, A., Sprung, M. & Salditt, T. (2014). *Acta Cryst.* A**70**, 552–562.

[bb46] Williams, G. J., Quiney, H. M., Peele, A. G. & Nugent, K. A. (2007). *Phys. Rev. B*, **75**, 104102.

